# RISK FACTORS FOR FALL-RELATED MILD TRAUMATIC BRAIN INJURIES AMONG OLDER ADULTS: A SYSTEMATIC REVIEW

**DOI:** 10.1093/geroni/igad104.3577

**Published:** 2023-12-21

**Authors:** Prince Okrah, Shafer Tharrington, Isaac Shin, Aaron Wagoner, Katelyn Woodsmall, Deborah Jehu

**Affiliations:** Augusta University, Augusta, Georgia, United States; Augusta University, Augusta, Georgia, United States; Augusta University, Augusta, Georgia, United States; Augusta University, Augusta, Georgia, United States; Augusta University, Augusta, Georgia, United States; Augusta University, Augusta, Georgia, United States

## Abstract

Mild traumatic brain injuries (mTBI) are commonly undiagnosed, which delays treatment and recovery. About 80% of older adults’ mTBIs stem from falls, yet factors driving fall-related mTBIs are unclear. This study aimed to systematically review risk factors for fall-related mTBIs among older adults. We used the Preferred Reporting Items for Systematic Reviews with Meta-Analysis protocol and the Meta-Analysis of Observational Studies in Epidemiology guidelines (Prospero ID: CRD42023377847). Our scope included prospective studies analyzing risk factors for fall-related mTBI in adults ≥ 60 years. The primary outcome measure was the relative risk for fall-related mTBIs, and secondary outcomes were: fall rate, total falls, and the number of fallers and non-fallers in those with and without a mTBI. On November 4, 2022, we searched: CINAHL Plus, and Health Source: Nursing Academic Edition, Nursing & Allied Health Database, PubMed, SPORTDiscus, and Web of Science. We conducted a grey literature search by examining reference lists of relevant studies. Two authors screened articles, and one author adjudicated any disagreements. Two authors would have assessed the reporting quality using the Strengthening the Reporting of Observational Studies in Epidemiology checklist, and the risk of bias using the Joanna Briggs Institute Prevalence Critical Appraisal tool. The database search yielded 410 articles; after deduplication, 395 titles and abstracts were screened, and 66 articles underwent full-text review. Overall, no studies were eligible. Fall-related mTBIs can have devastating consequences. There is an urgent need to identify risk factors for fall-related mTBIs in older adults to improve screening procedures and therapeutic intervention.

